# An OX40L mRNA vaccine inhibits the growth of hepatocellular carcinoma

**DOI:** 10.3389/fonc.2022.975408

**Published:** 2022-10-13

**Authors:** Zhuoya Deng, Hao Yang, Yuying Tian, Zherui Liu, Fang Sun, Penghui Yang

**Affiliations:** ^1^ Medical School of Chinese PLA, Beijing, China; ^2^ Faculty of Hepato-Pancreato-Biliary Surgery, Institute of Hepatobiliary Surgery, The First Medical Center, Chinese PLA General Hospital, Beijing, China; ^3^ Peking University 302 Clinical Medical School, Peking University, Beijing, China

**Keywords:** OX40L, mRNA cancer vaccine, hepatocellular carcinoma, immunotherapy, lipid nanoparticle

## Abstract

mRNA cancer vaccines show therapeutic potential for malignant tumors, including hepatocellular carcinoma (HCC). We optimized and synthesized stable mRNA encoding costimulator Oxford 40 ligand (OX40L). For systemic delivery, OX40L mRNAs were loaded into lipid nanoparticles (LNPs). The expression and costimulatory effects of OX40L were investigated *in vitro*. OX40L was expressed on the cell surface and costimulated T cells. *In vivo*, intratumoral injection of LNPs encapsulating OX40L mRNAs significantly reduced tumor growth and increased the survival of mice bearing H22 tumors. Importantly, CD4+ and CD8+ T cells were significantly increased in the OX40L mRNA group *in vivo*. Taken together, our findings provide a promising clinical strategy for immunotherapy for HCC using mRNA vaccines.

## Introduction

Hepatocellular carcinoma (HCC), the fourth-leading cause of cancer mortality worldwide, accounts for ~90% of liver cancers (1). Because of recurrence and poor prognosis, the global burden of HCC is increasing. Due to the limited efficacy of conventional therapy, the 5-year survival rate of HCC is poor (2). Therefore, novel, effective therapies are needed. Cancer immunotherapies, such as immune checkpoint inhibitors (ICIs), have emerged as novel pillars of cancer therapy. In HCC, early clinical trials with aPD-1/aPD-L1 and aCTLA-4 monotherapies have shown promising results with encouraging survival and safety data (3–6). Unfortunately, the ICIs used in those studies were either not effective or were only partially effective for a substantial portion of cancer patients (7). Novel treatments that circumvent this poor response or resistance are urgently needed.

Cancer vaccines have attracted attention because of their safety and efficacy. mRNA vaccines have been explored for the treatment of various diseases since the 1990s (8), including coronavirus disease 2019 (9–11). They may have therapeutic potential for cancer. Compared to DNA and protein vaccines, mRNA vaccines are safe, targeted, and are simple to manufacture, among other advantages (12). An mRNA vaccine is a modified mRNA molecule delivered into the cytoplasm and transcriptionally expressed using free nucleotides to generate the required protein. mRNA tumor vaccines are theoretically capable of encoding any protein, including tumor antigens and immune factors. To date, cancer vaccines have been composed of tumor-associated antigens (TAAs), tumor-specific antigens (TSAs), and immunostimulants. However, the large number of nucleases in the internal and external environments and the ability of biological macromolecules such as serum proteins to interact nonspecifically with nucleic acids could induce dissociation or aggregation of mRNAs, which are hydrophilic, negatively charged macromolecules with a limited ability to span membranes. Thus, the instability of mRNA vaccines is a key technical challenge that limits their clinical application. Although multiple delivery systems are being investigated, lipid nanoparticles are typically used to deliver mRNA vaccines to target cells.

Oxford 40 ligand (OX40L), a tumor necrosis factor receptor, is a homologous ligand of OX40 (13). The OX40–OX40L interaction acts as a T-cell costimulatory molecule, enhancing T-cell activation and proliferation to increase the antitumor immune response (14). OX40L is a promising target for mRNA cancer vaccines because its effective role compared to other molecules' mRNA encoding OX40L has been evaluated in a clinical trial (NCT03323398) and a research study (15). An mRNA vaccine encoding OX40L (mRNA-2416) for ovarian cancer has entered a phase II trial (NCT03323398), and a triplex mRNA vaccine encoding OX40L/IL23/IL36γ (mRNA-2752) for advanced solid tumors and lymphomas has entered phase I (NCT03739931). A triplex mRNA vaccine can trigger substantial immune cell recruitment into tumors, enabling their destruction (16). Notably, triplex mRNA vaccines have been evaluated mainly using MC38 cancer cells. The mechanism of action of the single OX40L mRNA vaccine and its effect on HCC progression are unknown. However, it has several disadvantages, such as instability and uncertain secondary structure. Therefore, we explored the mechanism of action of OX40L mRNA in HCC. We optimized the OX40L sequence and synthesized a lipid nanoparticle-encapsulated OX40L mRNA cancer vaccine. We assayed OX40L expression in various HCC cell lines. Subsequently, we evaluated the mRNA-loading capacity of the lipid nanoparticle vaccine and its antitumor effect and immune-activation mechanisms *in vitro* and *in vivo*. The data provide a theoretical basis for the development of an mRNA vaccine against liver cancer.

## Materials and methods

### Cell lines and strains

HCC cell lines (Huh7, HepG2, Hep3B, and H22) and the HEK293T human endothelial kidney cell line were obtained from the ATCC. Huh7, HepG2, H22, and HEK293T cells were maintained in high-glucose DMEM and Hep3B cells were maintained in MEM. All culture media were supplemented with 10% fetal bovine serum and 1% penicillin- streptomycin. Cells were cultured at 37 in a 5% CO2 humidified atmosphere. Competent DH5α cells for plasmid amplification were purchased from Tiangen.

### Optimization of the OX40L sequence and linearization of template DNA

GenBank was used to query and screen the target sequence open reading frame of hOX40L (NM_003326). The Kozak sequence was added to the 5′ end (17, 18). To optimize the whole target sequence, codon usage bias was adjusted and the average GC content was evaluated (19, 20). The sequence was constructed on pcDNA3.1, and the ligated enzyme cleavage sites were *BamHI* and *XbaI* . The designed OX40L recombinant pcDNA3.1 plasmid was optimized and synthesized at Biotech Bioengineering (Shanghai) Co.

The plasmid with the target fragment was extracted and the linearized template OX40L-pcDNA3.1 was obtained by monoenzymatic cleavage of the target plasmid by *XbaI* in a water bath at 37°C for 30 min, followed by column purification using a DNA purification kit. Agarose gel electrophoresis was performed to determine whether the linearized plasmid was complete, and whether the concentration and absorbance were eligible was evaluated using a nucleic acid concentration assay.

### *In vitro* transcription and transfection of OX40L-mRNA

OX40L mRNA was produced using the T7 polymerase-based *in vitro* transcription method (Novoprotein, GMP-E121). The linearized template OX40L-pcDNA3.1 was added to a 20-µL *in vitro* transcription reaction system at 1 µg and placed in a water bath at 37°C for 2 h; one third of the UTP was replaced with N1-methylpseudo-UTP to improve translation efficiency (21, 22). Next, the template strand was removed by adding DNase I (Novoprotein, GMP-E127) and placing the sample in a water bath for 15 min. Cap1 or ARCA cap structures were synthesized using the cowpox cap system or ARCA cap system, and poly(A) tails of >100 bases were added using *Escherichia* coli poly(A) polymerase (Novoprotein, GMP-M012). Stable OX40L-mRNA by these modifications, After these modifications, stable OX40L mRNA was subjected to column purification using an RNA purification kit (NEB, T2040). Finally, the absorbance and concentration of the mRNA were determined using a nucleic acid concentration assay and agarose gel electrophoresis. The purified OX40L-mRNA was transfected into *in vitro* cultured HEK293T cells, Hep3B cells, Huh7 cells and H22 cells using Lipofectamine 3000 (Invitrogen), and LNPs. Cells were collected at 3, 6, 12, 24, and 48 h.

### LNP-OX40L-mRNA preparation

Liposome components were dissolved in ethanol as the organic phase at molar ratios of 50:10:38.5:1.5 (DLin-MC3-DMA : DSPC:cholesterol:DSPE-PEG2000) (23, 24). OX40L mRNA was dissolved in 50 mM acetic acid buffer (pH 4.3) at 200 µg/mL as the aqueous phase. The organic and aqueous phases were passed through a microfluidic chip at a flow volume ratio of 1:3 to obtain LNP-OX40L mRNA. The encapsulation rate and particle size were evaluated *via* electron microscopy.

### RT-qPCR

To evaluate the effect of LNP-OX40L-mRNA on cell transcription, the HEK293T cells were cultured with OX40L-mRNA for 3, 6, 12 or 24 h. Total RNA was isolated using an R1200RNA Isolation Kit (Solarbio, Beijing, China) according to the manufacturer’s instructions. cDNA was synthesized using a cDNA Synthesis Mix Kit (KR118, Tiangen, Beijing, China). Quantitative real-time polymerase chain reaction was performed using SYBR Green qPCR PreMix (FP207, Tiangen, Beijing, China). OX40L gene expression was normalized to that of GAPDH. The ΔΔCT method was used to analyze OX40L gene expression. The primer sequences used were: forward primer OX40L 5′-GCGTGATCATCAACTGCGAC-3′ and reverse primer OX40L 5′-TGTTCACGCTCCTCACCTTC-3′; forward primer GAPDH 5′-ACAACTTTGGTATCGTGGAAGG-3′ and reverse primer GAPDH 5′-GCCATCACGCCACAGTTTC -3′.

### *In vitro* biological activity of OX40L

Blood samples were obtained from three healthy individuals, and PBMCs were isolated by density gradient centrifugation using Ficoll-Hypaque. Next, the PBMCs were washed with PBS twice followed by centrifugation at 2000 rpm for 10 min. Isolated PBMCs were cultured in RPMI 1640 medium with 10% FBS at 37 under 5% CO2 for 24 h. T cells were purified from human PBMCs by negative selection using MACS beads (Miltenyi Biotec) according to the manufacturer’s instructions. The T cells were stimulated with an anti-CD3/CD28 antibody and cocultured with Hep3B cells transfected with OX40L-mRNA for 24 h. The IL-2 level in supernatant was measured using an IL-2 Precoated ELISA Kit (Dakewe Biotech, 1110202) according to the manufacturer’s instructions (25). This study was approved by the Ethics Committee of the Fifth Medical Center of Chinese PLA General Hospital.

### Western blotting analysis

Control, original, and optimized mRNA sequences were transfected into HEK293T cells for 24 h, and the cells were harvested using 0.25% EDTA-Trypsin. HepG2, Huh7, Hep3B, and H22 cells transfected with optimized OX40L mRNA were also harvested using 0.25% EDTA-Trypsin. Total protein was extracted using RIPA buffer (Solarbio, R0010). Protein concentrations were determined using a BCA Protein Assay Kit according to the manufacturer’s instructions. Next, 10 µg protein was resolved by 10% SDS-PAGE, transferred onto a polyvinylidene fluoride membrane, and nonspecific binding was blocked using 5% BSA blocking buffer (Solarbio, SW3015). Subsequently, the blot was incubated overnight with a rabbit anti-OX40L antibody (1:1500 dilution, CST, 14991) and mouse anti-β-actin antibody (1:1500 dilution, CST, 3700) in protein standard solution at 4 . The secondary antibodies were HRP-conjugated goat anti-rabbit (1:5000 dilution) and HRP-conjugated goat anti-mouse (1:5000 dilution) antibodies. Bands were visualized using an ECL detection system.

### Immunofluorescence staining and microscopy

For immunofluorescence staining, HEK293T cells were seeded on glass coverslips pretreated with TC (Solarbio) (26). After incubation for 24 h to reach 75% confluence, the cells were transfected with OX40L mRNA. The controls were cells treated with non-coding mRNA. Next, the cells were cultured in fresh medium for 24 h, harvested, and fixed with 4% paraformaldehyde for 30 min, followed by three washes with PBST. Subsequently, the HEK293T cells were blocked with 5% BSA blocking buffer. Immunostaining was carried out using a rabbit anti-OX40L antibody (1:200, Abcam, ab263910) in PBST at 4 overnight. The coverslips were washed three times with PBST and incubated with Alexa Fluor^®^ 488-conjugated goat anti-rabbit IgG H&L (1:250, Abcam, ab150077) for 1 h at room temperature. Nuclei were counterstained with DAPI (Solarbio, C0065). Cells were mounted on slides and imaged under a confocal laser scanning microscope.

### *In vivo* assays

Four- to six-week-old female Kunming or BALB/C mice were purchased from SPF (Beijing) Biotechnology Co. and housed in an SPF environment with regular watering and feeding. As a tumor-bearing mouse model, H22 cells were preinoculated intraperitoneally into Kunming mice. One week later, cells with ascites were harvested and 5 × 10^6^ H22 cells were subcutaneously injected into the left flank of the mice. When the tumor volume reached 80 mm3, the mice were randomly divided into two groups. Those in the OX40L group underwent intratumoral injection of 11.5 µg LNP-OX40L mRNA and those in the control group received an equal amount of non-coding mRNA. Intratumoral injections were performed six times at 3-day intervals. The efficacy of mRNA-expressing OX40L in the liver tumor model was assessed by continuous measurement of tumor size, and tumor tissues were collected and assessed for OX40L expression *via* ELISA and FACS. The effects of OX40L expression and activation on immune cells at tumor sites were assessed by flow cytometry. The endpoints of the study were animal death or a tumor reaching 2000 mm^3^ in size. Tumors were isolated and weighed, and tumor volume was calculated as (tumor length × tumor width^2^× 0.5), Kaplan-Meier survival curves were plotted after 42 days. Procedures involving animals were approved by the Animal Welfare and Ethics Committee of the Fifth Medical Center of the PLA General Hospital.

### Fluorescence-activated cell sorting

To monitor transfection efficiency *in vitro*, HEK293T cells were harvested after 24 and 48 h and washed twice with PBS. Next, the cells were stained with a PE anti-human CD252 (OX40L) antibody following the manufacturer’s instructions. After incubation in the dark for 30 min at room temperature, the cells were fixed with PBS containing 1% paraformaldehyde and subjected to fluorescence-activated cell sorting analysis.

To analyze T-cell activation and immune-cell phenotype *in vivo*, spleens were harvested from H22 tumor-bearing mice, minced, and rinsed in 40-µm mesh cell strainers filled with PBS to harvest cell suspensions. These cells were preactivated using Cell Activation Cocktail (BioLegend, 423303) for 4 h. The cells were stained with FITC anti-mouse CD4, PerCp-CY5-A anti-mouse CD8 and BV510 anti-mouse CD69 antibodies at room temperature for 30 min. After staining the extracellular antibodies, PE anti-mouse IFN-γ was stained at room temperature for 20 min. After washing with PBS, samples were fixed with PBS containing 1% paraformaldehyde and subjected to flow cytometry; the data were analyzed and visualized using FlowJo software.

### Histological and biochemical assessment

For immunohistochemistry, tumor samples were collected, embedded in paraffin, sectioned, and stained using CD4, CD8, and Ki67 antibodies. For histological assessment, heart, liver, spleen, lung, kidney, and brain samples were processed as above. Hematoxylin and Eosin (H&E) staining was performed to assess the effects of LNP-OX40L mRNA on other tissues. Stained liver sections were visualized under a microscope.

### Statistical analysis

Statistical analysis was performed using SPSS 25.0 and GraphPad Prism software. Data are expressed as medians ± minimum to maximum or means ± SDs. Between-group comparisons were performed using the *t*-test. Comparisons of multiple groups were performed using one-way ANOVA with Dunnett’s multiple comparison *post hoc* test. A *p* value < 0.05 was considered indicative of significance. Differences are labeled * *p ≤*0.05, ** *p ≤*0.01 and *** *p ≤*0.001. The sample size required for each experiment was determined to be the minimum necessary for statistical significance based on common practice in the field. Similarities in the variances of each statistically compared group were verified by *F*-test.

## Results

### Characterization and *in vitro* transcription of OX40L mRNA

**Figure 1:**
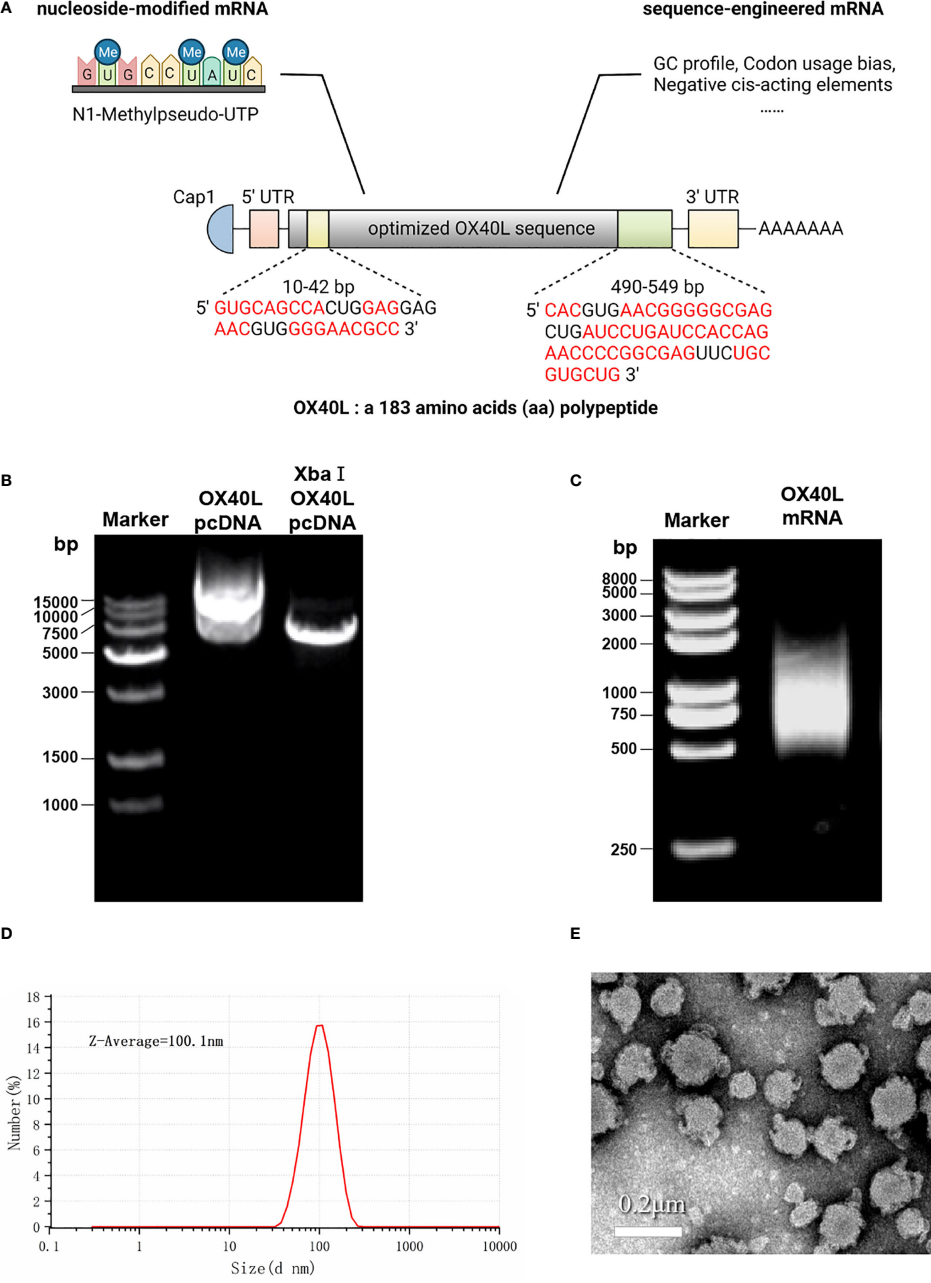
Optimization and characterization of encapsulated OX40L mRNA. **(A)** Structure of the optimized mRNA. Codons in red are optimized. **(B)** Identification of recombinant plasmid and restriction-digested plasmid by gel electrophoresis 1) 15000 bp marker, 2) 600 ng OX40L-pcDNA3.1 and 3) 200 ng *Xba I* -digested OX40L-pcDNA3.1. **(C)** Electrophoretic profile of mRNA 4) 2000 bp marker; 5) 2 ug OX40L-mRNA. **(D)** Size distribution of OX40L mRNA-LNP. **(E)** TEM image of OX40L mRNA-LNP (scale bar: 200 nm).

To develop a potential OX40L mRNA vaccine for HCC, we found the original sequence of OX40L using GenBank. The Kozak sequence GCCACCAUGG was added to the 5′ end to increase the translation efficiency. Codon usage bias adjustment increased the Codon Adaptation Index value from 0.54 to 0.93. The average GC content was adjusted from 46.3% to 55.7% and unfavorable peaks were removed (**Table 1**). A schematic of the mRNA is shown in **Figure 1**. We extracted the synthesized recombinant pcDNA3.1 plasmid, and gel electrophoresis detected a circular plasmid of ~6000 bp. The recombinant plasmid was cut once by Xba I, and a linearized template of ~6000 bp was identified by gel electrophoresis (**Figure 1B**). After *in vitro* transcription and addition of a Cap 1 and poly (A) tail, gel electrophoresis indicated that the mRNA band size was 750–1000 bp. Synthetic mRNA was quantified using a nucleic acid concentration assay (**Figure 1C**).

**Table 1:** Sequence optimization score of the optimized and original sequences.

	CAI	GC%	Negativecis-acting elements	Negative repeat elements
Optimized OX40L sequence	0.93	55.7	0	0
Original OX40L sequence from NM_003326	0.54	46.3	1	0

^a^Codon usage bias was adjusted to fit the highest expression profile of the target host. A CAI (Codon Adaptation Index) of 0.8 - 1.0 is regarded as good for high expression.

^b^Repeated area in the original sequences were removed to avoid stem-loop structures in mRNA and facilitate the synthesis process.

^c^Undesired motif including restriction enzyme site to be used in sub-cloning and negative cis-acting sites were modified.

^d^Whole sequence was fine-tune to increase the translation efficiency and prolong the half-life of mRNA.

### Optimized OX40L mRNA is expressed on the HCC cell membrane

**Figure 2:**
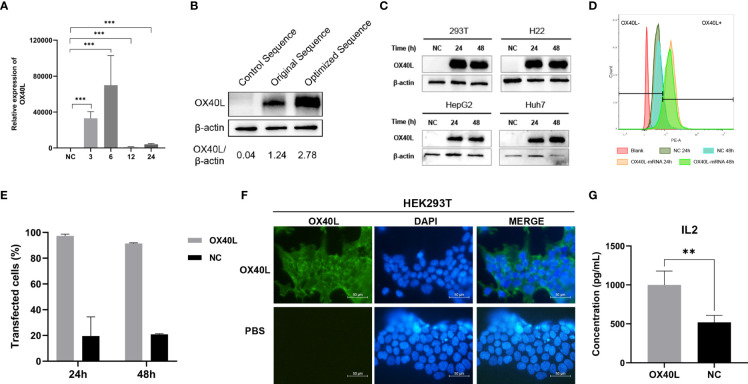
Identification, localization, and function of OX40L-mRNA protein. **(A)** Effect of OX40L mRNA-LNP according to RT-qPCR **(B)** Western blotting analysis of protein expression from the optimized and original sequences in HEK293T cells. **(C)** Western blotting analysis of optimized OX40L mRNA transfected into HCC cell lines. **(D)** Flow cytometry analysis of transfection efficiency in HEK293T cells at 24 h and 48 h. **(E)** Quantitative analysis of **(C)**. **(F)** Immunofluorescence images of transfected HEK293T cells (scale bars: 100μm). **(G)** ELISA of the supernatant IL2 levels in cocultures of lymphocytes with OX40L mRNA transfected Hep3B cells. Statistical significance was calculated *via* t test. **P < 0.01; ***P < 0.001.

Transfection of the optimized OX40L mRNA increased OX40L expression compared to the original sequence (**Figure 2**). RT-qPCR showed that OX40L mRNA was transfected in the cells. Western blotting identified OX40L protein in all HCC cell lines. Flow cytometry showed that protein expression peaked at 24 h. Immunofluorescence localized the protein to the cell membrane. Therefore, OX40L mRNA was expressed on the cell membrane *in vitro*.

### OX40L enhances the activation of T cells prestimulated with CD3/CD28

OX40L acts as a costimulator in tumor immunity. Therefore, we established a coculture model of T cells and Hep3B cells transfected with OX40L mRNA *in vitro* to investigate immune costimulation in HCC. ELISA showed that coculture of prestimulated immune cells with Hep3B cells transfected with OX40L mRNA increased IL2 production by T cells; CD3/CD28 generates the first stimulation, which is enhanced by OX40L (**Figure 2G**).

### Intratumoral injection of OX40L mRNA-LNP inhibits tumor progression

**Figure 3:**
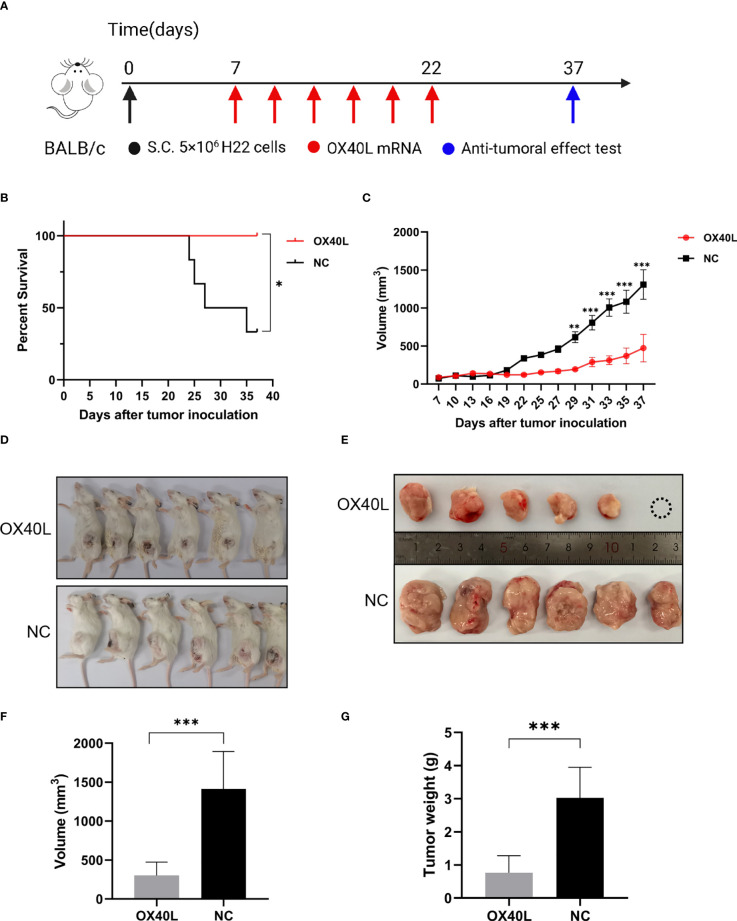
*In vivo* efficacy of intratumoral therapy with OX40L mRNA. **(A)** Timeline for treatment of H22 tumor-bearing mice. **(B)** Kaplan-Meier analysis of mouse survival after treatment with OX40L-mRNA and non-coding mRNA; log-rank test, p =0.0183. **(C)** Growth of subcutaneous tumors growth curves (median ± interquartile range [IQR]) of each group treated with OX40L-mRNA and the control. Two-way repeated-measures ANOVA using the Sidak multiple-comparisons test, **p≤ 0.01, ***p≤ 0.001, compared to non-coding mRNA. **(D)** Images of mice from the two groups at day 37. **(E)** Tumors; white circle, no tumor. **(F)** Tumor volume at day 37. Statistical significance was calculated *via* t test. ***p ≤ 0.001. **(G)** Tumor weight at day 37. Statistical significance was calculated *via* t test. ***p≤0.001.

**Figure 4:**
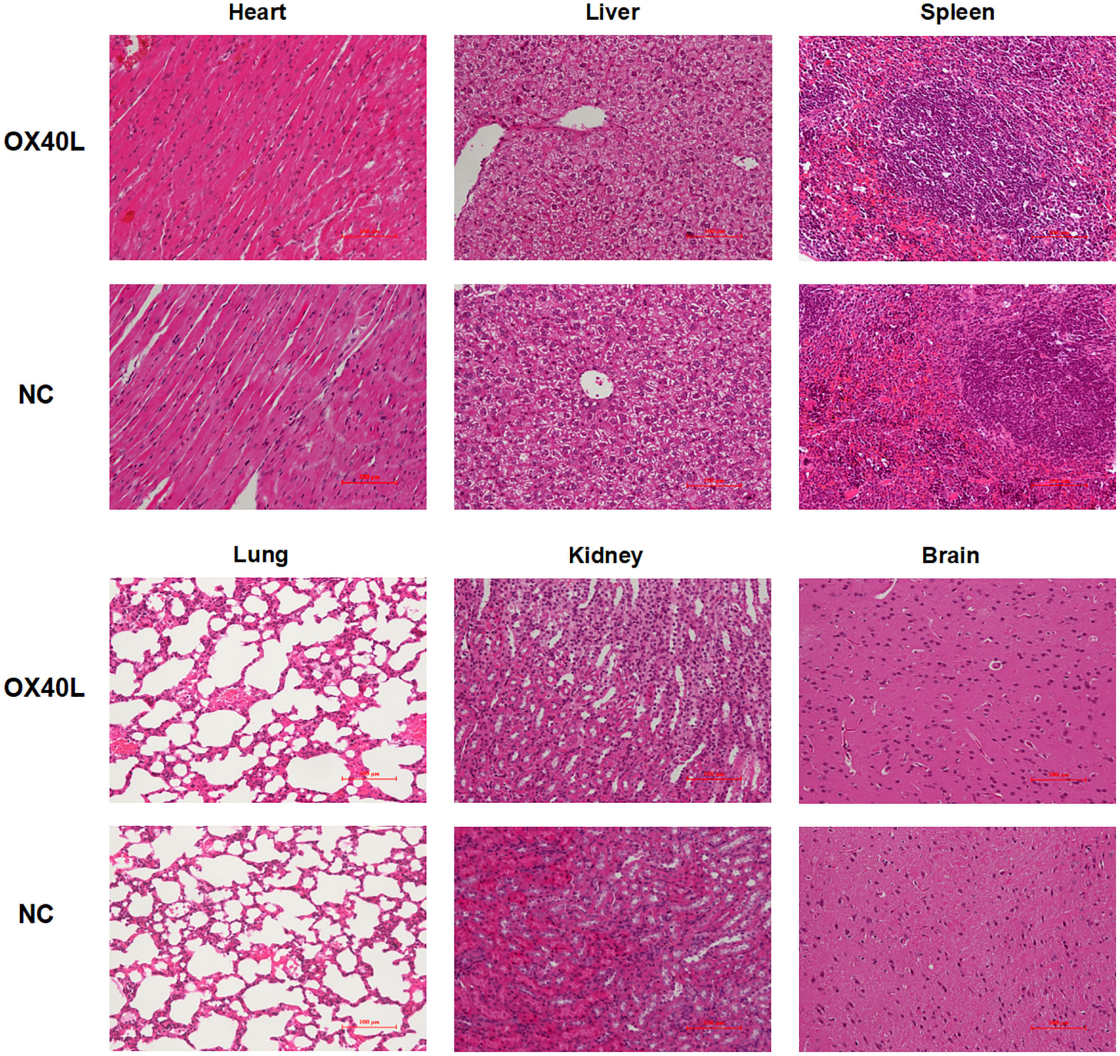
Organ toxicity of OX40L mRNA-LNP. Mice were euthanized at the end of treatment and their organs were removed. Heart, liver, spleen, lung, kidney and brain sections were stained with H&E to evaluate organ toxicity (scale bars: 100μm).

To enhance vaccine delivery, we encapsulated OX40L mRNA in lipid nanoparticles. The particle size of the encapsulated mRNA was 100.1 nm and its polydispersity index was about 0.08 (**Figure 1**). Electron microscopy showed that LNP encapsulated OX40L mRNA. To evaluate the delivery efficiency and therapeutic effect of OX40L mRNA-LNP, further we established an H22 tumor bearing model by intratumoral injection of OX40L mRNA-LNP. After six injections and 2 weeks of observation, the tumor size after intratumorally injected mRNA-OX40L was significantly smaller than that after intratumorally injected encapsulated non-coding mRNA (*p* ≤ 0.001; **Figure 3**). After treatment for 14 days, the mean tumor size was 1265.71 mm^3^ in the control group and 472.52 mm^3^ in the OX40L group. Survival curves are shown in **Figure 3C**. Mice were euthanized on day 37 to photograph the tumors. The mean weights of tumors in the control and OX40L groups were 3.02 and 0.77 g, respectively. In addition, H&E staining of heart, liver, spleen, lung, kidney, and brain tissues showed no significant differences between the two groups (**Figure 4**), implying that the OX40L mRNA vaccine was safe and did not harm tissues. Therefore, intratumoral injection of OX40L mRNA encapsulated in LNPs significantly inhibited the growth of H22 subcutaneous tumors.

### OX40L mRNA induces T cell activation *in vivo*

**Figure 5:**
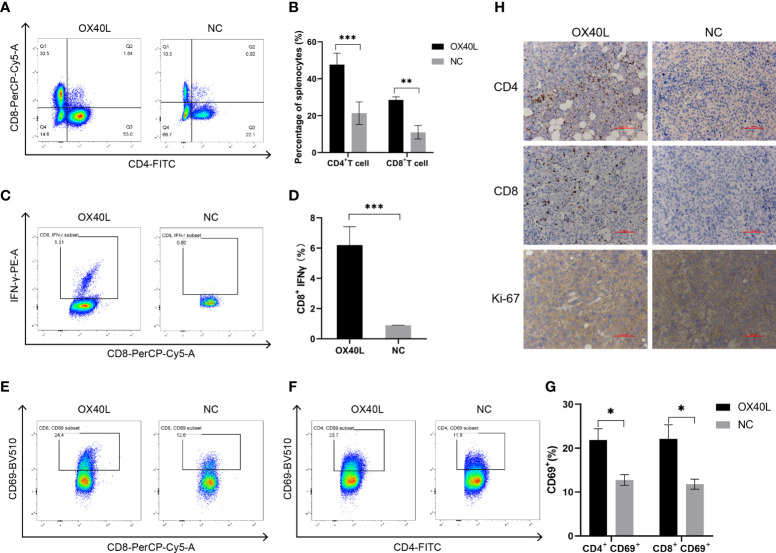
OX40L mRNA elicited lymphocyte activation in the H22 tumor bearing mice. **(A, C)** T-cell activation by flow cytometry. CD4^+^ T cells and CD8^+^ T cells in the spleen of the H22 tumor-bearing BALB/c mice. **(B, D)** Quantitative analysis of the proportions in **(A**, **C)**. Statistical significance was calculated *via* t test. **P ≤ 0.01, ***P ≤ 0.001. **(E, F)** Representative FACS profiles of CD69^+^ CD8^+^ and CD69^+^ CD4^+^ activated T cells. **(G)** Results of quantitative analysis of the proportions in **(E, F)**. Statistical significance was calculated *via* t test. *P ≤0.05. **(H)** Immunohistochemistry of the mechanisms of action of OX40L mRNA in subcutaneous H22 tumors (scale bars: 100μm).

Flow cytometry showed that the percentages of CD4^+^ and CD8^+^ T cells in the OX40L group were significantly higher than that in the control group (**Figure 5**). In addition 7.06% of CD8^+^ T cells in the OX40L group expressed IFNγ protein, significantly more than in the control group. CD69^+^ is a marker of early activation of T cells and is implicated in their immune activities. Flow cytometry showed that the percentages of CD4^+^ CD69^+^ T cells and CD8^+^ CD69^+^ T cells in the OX40L group were 23.7% and 24.4%, respectively, higher than in the NC group (11.9% and 12.5%, respectively). Immunohistochemistry indicated greater infiltration of CD4+ and CD8+ T cells in the OX40L group than in the control group. In addition, ki67 staining was enhanced in the control group, indicating that OX40L suppressed tumor proliferation. Therefore, OX40L may induce immune effects by stimulating T cells and upregulating IFN-γ, thereby suppressing tumor-cell proliferation and inhibiting HCC progression.

## Discussion

Because of the lack of specific manifestations in the early stages, many patients have advanced HCC at the time of diagnosis. Current treatments are insufficiently efficacious against relapsed and refractory HCC (2). With the advances in tumor immunology techniques and molecular biology represented by mRNA vaccines, tumor immunotherapy offers new hope to patients. New and improved immunotherapeutic approaches with higher standard of safety and efficacy are being implemented in oncology. In this study, we evaluated immunomodulators that trigger immune responses and improve the immune microenvironment. The safe local delivery of mRNAs encoding different targets is an effective tumor immunotherapy modality.

mRNA cancer vaccines have been used against a variety of malignant tumors and encode a variety of target proteins, including TAAs, TSAs, and immunostimulants (27). In most early clinical trials, cancer vaccines targeted TAAs (28–30). With the development of second-generation sequencing and bioinformatics technologies, personalized mRNA cancer vaccines expressing TSAs are under development (31), as are broad-spectrum vaccines encoding immunostimulants that mobilize T-cell responses. OX40L is a type II transmembrane glycoprotein of molecular weight 34 kDa. OX40 conjugates with OX40L and transmits costimulatory signals to counteract the suppression of immune cells and stimulate effector T cells to perform proinflammatory functions (14). OX40-OX40L has been used as a target for immunotherapy in a variety of tumors. OX40L mRNA has demonstrated efficacy against, for example, ovarian and colorectal cancers (32–34) but it has not been evaluated in liver cancer. In this study, we optimized and synthesized OX40L mRNA to enhance OX40L expression (19). OX40L mRNA was expressed in several liver cancer cell lines and acted as a costimulatory molecule *in vitro*. We explored the antitumor effect and underlying mechanism of action of the OX40L mRNA vaccine *in vivo*.

Optimized mRNA was synthesized based on *in vitro* transcription. We added an miRNA-122 binding site to the 3′-UTR to reduce the effect of OX40L on normal hepatocytes (35). The Kozak sequence significantly enhances mRNA translation efficiency, so we added it to our vaccine (17, 36). We replaced part of the UTP with N1-methylpseudo-UTP to reduce its immunogenicity (21, 22). Western blotting and flow cytometry verified expression of OX40L, which was stable for 48 h. OX40L is a membrane-bound ligand expressed on the cell surface (37). Indeed, immunofluorescence showed that OX40L was expressed on the cell membrane. Costimulatory factors can induce IL-2 production and facilitate T-cell immunity (38). In this study, OX40L acted as a costimulatory factor to activate immune cells by enhancing IL-2 expression in cocultures of OX40L-expressing Hep3b cells and isolated T cells.

We used conventional MC3-LNP encapsulated mRNA for *in vivo* delivery (39). The average diameter of the encapsulated mRNA was 100.1 nm according to dynamic light scattering analysis. The polydispersity index was ~0.08, indicating uniform distribution of the mRNA after encapsulation (40). The electron micrographs visually corroborated the morphology and size of the encapsulated mRNA. Subsequently, the encapsulated mRNA was injected intratumorally into mice and showed effective anti-tumor results. Local delivery of this mRNA by intratumoral injection increased the CD4^+^ and CD8^+^ T cell populations in the spleen and tumor, promoting tumor immune infiltration and tumor suppression. The higher populations of both CD4^+^ CD69^+^ and CD8^+^ CD69^+^ T cells showed the early activation of T cells. IFNγ release by CD8^+^ T cells may be responsible for immune killing of tumors. The costimulatory effect on T-cell activation is consistent with Mendel et al. (41). Ki67 immunohistochemistry showed that injection of hepatocyte tumors with OX40L mRNA resulted in low malignancy and a good prognosis. These immunological results are consistent not only with the tumor volume and weight but also with previous reports of tumor suppression by OX40/OX40L (42–44). Examination of pathological sections showed that vaccination did not cause organ damage, possibly because local injections overcome the toxicity and low concentration at the tumor site associated with systemic administration (45, 46). Therefore, we speculate that OX40L mRNA is a safe and effective mRNA vaccine against HCC. Moreover, overexpression of OX40L in the tumor microenvironment or in tumor cells can enhance therapeutic efficacy and reduce off-target effects of the vaccine.

In summary, intratumoral injection of OX40L mRNA in LNPs has therapeutic potential for HCC. Next the efficacy of OX40L mRNA in LNPs needs to be evaluated using a variety of other models. Mice depleted of CD4 or CD8 T cells will be used to perform mechanistic investigations. Importantly, OX40L is but one of many human immunomodulatory factors. Therefore, combining mRNA vaccines with immunomodulatory factors, potentially several such factors, could reverse intratumoral immunosuppression and elicit a curative antitumor immune response. OX40L mRNA cancer vaccine, alone or in combination with other cancer therapies, has potential for the immunotherapy of a variety of tumor types.

## Data availability statement

The original contributions presented in the study are included in the article/ Supplementary Material. Further inquiries can be directed to the corresponding author.

## Ethics statement

The studies involving human participants were reviewed and approved by Ethics Committee of the Fifth Medical Center of Chinese PLA General Hospital. The patients/participants provided their written informed consent to participate in this study. The animal study was reviewed and approved by Animal Welfare and Ethics Committee of the Fifth Medical Center of the PLA General Hospital.

## Author contributions

ZD conducted the experiments, analyzed the data and wrote the manuscript. HY, YT, and FS analyzed the data. ZL completed the figures. PY designed the experiments and supervised the work. All authors read and approved the submitted version.

## Conflict of interest

The authors declare that the research was conducted in the absence of any commercial or financial relationships that could be construed as a potential conflict of interest.

## Publisher’s note

All claims expressed in this article are solely those of the authors and do not necessarily represent those of their affiliated organizations, or those of the publisher, the editors and the reviewers. Any product that may be evaluated in this article, or claim that may be made by its manufacturer, is not guaranteed or endorsed by the publisher.
